# Repurposing public sarcoma multi-omics for neoantigen discovery

**DOI:** 10.1007/s00262-026-04395-y

**Published:** 2026-04-21

**Authors:** Panagiotis Mantas, Karen A. Krogfelt

**Affiliations:** 1 Department of Health Technology, Technical University of Denmark, 2800 Kongens Lyngby, Denmark; 2 Department of Science and Environment, Roskilde University, 4000 Roskilde, Denmark

**Keywords:** RNA-guided neoantigens, Complex karyotype sarcomas, Immunogenomics, Legacy sequencing

## Abstract

**Background:**

Soft tissue sarcomas, particularly complex karyotype sarcomas (CKS), are characterized as “immunologically cold” malignancies driven by structural instability rather than a high tumor mutational burden (TMB). Public “legacy” cohorts are a useful resource to uncover immunotherapy biomarkers. This study used the whole-exome sequencing (WES) and RNA-sequencing of CKS patients, to overcome technical limitations and to identify and prioritize neoantigens.

**Methods:**

The systematic immunogenomic reanalysis was performed on a landmark cohort of CKS patients (Kim et al., 2018) with a custom bioinformatics workflow which was developed to uncover interpretable immunogenomic signals. This approach consisted of: (1) defining a quality-controlled “callable territory” and normalizing TMB metrics, respectively; (2) utilizing RNA-seq not only for expression filtering but as an orthogonal validation check for variant transcription and to distinguish functional amplifications from technical depth artifacts; and (3) applying a multi-modal epitope prediction pipeline to identify and prioritize high-affinity neoantigens derived from both somatic SNVs, indels and expressed gene fusions.

**Results:**

The reanalysis shows that standard genome-wide metrics frequently underestimated the immunogenic potential. Normalizing the TMB refined quantitative mutation burden estimates and improved interpretation of low-coverage samples without essentially changing the overall cohort classification. Furthermore, integration of transcriptomic data facilitated the recovery of actionable targets in “low-TMB” tumors. A subset of fusion-derived peptides demonstrated predicted binding affinities competitive with SNV-derived candidates.

**Conclusion:**

This study illustrates that technically constrained multi-omic datasets can be systematically re-analyzed to identify potential therapeutic targets. These data argue for looking beyond aggregate biomarkers; patient-specific, expressed neoepitopes may exist even in sarcomas typically described as immunologically “cold”.

**Supplementary Information:**

The online version contains supplementary material available at 10.1007/s00262-026-04395-y.

## Introduction

Soft tissue sarcomas are rare cancers with diverse biology. They have historically shown limited responsiveness to immune checkpoint blockade (ICB), particularly when compared with “hot” tumors such as melanoma or lung cancer [1, 2]. Standard biomarkers, such as tumor mutational burden (TMB), fall short on predicting immunotherapy response in cancers such as sarcomas because they overlook non-SNV neoantigen sources [3–5]. This metric is not well suited for sarcomas, which are broadly divided into two genomic classes: “Simple Karyotype” sarcomas (driven by specific translocations like EWSR1-FLI1) and “Complex Karyotype” sarcomas (CKS), which are driven by chaotic copy-number alterations and structural rearrangements rather than high SNV loads [6]. Thus, CKS tumors are frequently characterized as “immunologically cold”, despite possessing significant neoantigen potential driven by high genomic instability, copy-number alterations and DNA damage response (DDR) pathway defects [7–9]. Personalized vaccines (or shared neoantigens) remain still a promising frontier [2, 10–14]. Identifying immunogenic targets within this structural chaos could open new therapeutic paths for patients who currently have few options.

While a handful of genomic studies in sarcoma have explicitly evaluated neoantigens to inform survival associations or support vaccine design [1, 15], most large-scale paired WES and RNA-seq efforts have pursued different objectives. Cohorts have primarily focused on pinpointing core molecular drivers (e.g., PI3K/mTOR vulnerabilities in osteosarcoma or chromatin remodeling mutations in undifferentiated pleomorphic sarcoma (UPS)), defining molecular subtypes through integrated clustering as seen in rhabdomyosarcoma classification [7, 16, 17]. These analyses prioritized differentiation programs, copy-number variations (e.g., CDK4/RB1 signatures in Complex Karyotype Sarcoma), and transcriptional states to nominate drug targets (like PDGFR) and explain disease biology rather than performing systematic neoantigen discovery or immune-focused tumor characterization [18]. Yet these same datasets contain the exact raw data required for immunogenomics: somatic variant calls, expression evidence, and immune context. As a result, neoantigen-level information in these cohorts has not been systematically extracted. The raw sequencing is already public; reanalyzing it with current neoantigen prediction and prioritization workflows can uncover immunogenic determinants that were outside the scope of the original driver- and subtype-centric analyses, possibly yielding actionable vaccine targets without new patient samples.

To bridge this translational gap, a systematic immunogenomic reanalysis was performed on the Kim et al. CKS cohort using a state-of-the-art bioinformatics framework [18]. By integrating thorough quality control measures, which successfully recapitulated the original molecular classifications (e.g., CDK4-amplified subtypes), this study established a foundation for extending the study’s scope beyond driver identification. The presented approach specifically maps the neoantigen landscape across distinct genomic architectures, contrasting purported “Immunogenic Hotspots” against the traditionally viewed “Immunogenic Deserts” of complex karyotype sarcomas, to isolate overlooked vaccine candidates.

This is the first comprehensive profiling of the SNV- and fusion-derived neoantigen landscape in this specific CKS cohort. By applying a multi-modal epitope prediction pipeline, the results revealed that even these phenotypically “cold” tumors, often excluded from immunotherapy trials, harbor high-affinity, patient-specific targets born from their profound genomic instability.

## Materials and methods

### Data acquisition

Raw whole-exome sequencing (WES) and RNA-seq paired-end FASTQ files were retrieved from the European Nucleotide Archive (ENA) under the accession number PRJEB23898 and PRJEB24352. These data correspond to the cohort originally described by Kim et al. (2018), comprising 14 patients with complex karyotype soft tissue sarcomas (CKS) [18]. To enable deep multi-modal characterization while sustaining computational feasibility, a representative discovery set of six samples was selected, spanning the genomic subtypes defined in the original study. The remaining matched cases from the original cohort were processed identically, and their metadata and neoepitope summary results are provided in the Supplementary Materials. This discovery set comprised a hypermutator group characterized by elevated TMB and transition-enriched spectra (*n* = 3): UT05 (Undifferentiated Sarcomas (US)), FT13 (Myxofibrosarcoma), and LT02 (Leiomyosarcoma) and a low-SNV group (*n* = 3): UT01 (Undifferentiated Sarcomas, desert-like), UT08 (Undifferentiated Sarcomas, fusion associated), and LT01 (Leiomyosarcoma, amplification-driven). This grouping captures the major biological axes identified by Kim et al. (hypermutation vs. chromosomal stability, treatment-naïve vs. chemotherapy-exposed) and allows for a direct comparison of neoantigen landscapes across mechanistically distinct tumor subtypes.

### Genomic preprocessing & alignment

**Whole-exome preprocessing and alignment**: Raw WES FASTQ files were assessed with FastQC (v0.12.1) [19] to evaluate base quality, adapter contamination, and over-represented sequences. Illumina TruSeq adapter sequences and low-quality bases were trimmed using Cutadapt (v5.2) with a minimum Phred score of 20 and minimum post-trimmed read length of 50 bp [20].

Trimmed reads were aligned to the GRCh38/hg38 reference genome (updated from the original hg19 analysis to ensure compatibility with current genomic resources) using BWA-MEM (v0.7.19) [21]. PCR and optical duplicates were marked with Broad’s institute tool Picard MarkDuplicates (v3.4.0)(http://broadinstitute.github.io/picard). Following GATK Best Practices, the gnomAD resource was used for germline filtering in Mutect2 and GetPileupSummaries software.

Because the original study used hg19, all analyses in this study were performed de novo on GRCh38 to ensure compatibility with current annotation resources and HLA/neoantigen tools. The GRCh38/hg38 human reference genome and transcriptome data were downloaded from the Gencode Project website (https://www.gencodegenes.org/) [22].

**RNA alignment & quantification**: Tumor RNA-seq FASTQs underwent quality control with FastQC and adapter/quality trimming with Cutadapt as above. Reads were then processed through two complementary methods:

- STAR alignment used for fusion detection and expression-aware mapping. Reads were aligned to GRCh38 (with GENCODE transcriptome) using STAR (v2.7.10b) in two-pass mode with chimeric detection enabled to preserve fusion-relevant soft-clipped and discordant reads [23].
- Transcript-level quantification for expression filtering. In parallel, pseudo-alignment and transcript quantification were performed with Kallisto (v0.51.1) using GENCODE-derived transcript indices [24]. Transcript-level TPMs were summed to gene-level TPMs and later used to filter neoantigen candidates by expression.

### Somatic variant calling

Somatic SNVs and indels, including frameshift mutations were identified using GATK Mutect2 (v4.6.2.0) in tumor–normal mode, with each tumor BAM compared to its patient-matched germline WES BAM [25]. Default Mutect2 filters were applied via FilterMutectCalls, followed by additional stringent filters: a) PASS in Mutect2 filter field, i.e., passed all gatk tests such as contamination, strand bias, and generic bias. b) Tumor depth ≥ 10 × at the variant site.

Variants were functionally annotated with Ensembl VEP (v110). For the purpose of neoantigen prediction, downstream analysis was restricted to protein-coding missense SNVs and in-frame or frameshift indels, while synonymous variants and stop-gain mutations, were excluded. Stop-gain mutations introduce premature termination codons and therefore do not usually generate the altered peptide context modeled in standard NetMHCpan-based substitution/indel neoepitope prediction workflows. In addition, transcripts harboring premature stop codons are frequently subject to nonsense-mediated mRNA decay, reducing the likelihood of stable mutant transcript and protein expression. For these reasons, stop-gain variants are not prioritized for peptide-MHC binding in this study.

However, consistent with standard practice for mutation burden estimation, stop-gain mutations were retained as nonsynonymous events in the tumor mutational burden calculation. Because the dataset showed substantial capture bias with mean depth of 29.3x (range: 15.0–39.2x), TMB was normalized carefully using callable coding territory. The numerator was restricted to nonsynonymous somatic mutations (comprising missense, stop-gain, frameshift, and in-frame insertions/deletions) identified within the high-confidence intervals. To prevent TMB underestimation due to varying capture efficiency, the denominator was defined as the sample-specific callable coding territory, consisting of protein-coding CDS regions (GENCODE v49) that achieved a minimum of 10 × depth. This normalization avoided underestimating mutation load in poorly captured regions.

The MSI status for samples came directly from Kim et al.’s original hypermutator analysis, which identified MSI/MMR-associated mutational signatures and expression patterns in the hypermutator subset [18]. Transition-to-transversion (Ti/Tv) ratios were determined using bcftools stats (v1.17) based on somatic variants passing all quality filters (PASS) with a minimum tumor read depth of 10x, restricted to the exome capture regions.

### Fusion detection from RNA-seq

To identify immunogenic targets derived from structural variations, fusion transcripts were characterized using Arriba (v2.4.0) on STAR-aligned BAMs. Default Arriba annotations (blacklists for recurrent artifacts, read-through events, and immunoglobulin artifacts) were used [26]. High-confidence fusions meeting the following criteria were retained:

- At least 5 junction (split-read) reads and/or discordant read pairs
- Not present in Arriba’s internal blacklist (e.g., recurrent technical artifacts, rRNA, mitochondrial genes, or same-gene read-throughs).
- Only fusions classified as “high” confidence by Arriba were considered.
- In-Frame Events: Fusions were restricted to in-frame sequences to ensure a plausible open reading frame (ORF) for peptide generation.
- Expression metric: Fusion abundance was quantified using Fusion Fragments Per Million (FFPM), derived from fusion-specific support reads normalized by total mapped library depth.

### HLA class I typing

Patient-specific HLA class I genotypes (HLA-A, -B, -C; 4-digit resolution) were inferred from tumor RNA-seq using OptiType (v1.3.5), which utilizes coverage patterns across exons 2 and 3 to reconstruct the most likely allele combination. HLA typing was performed once per patient and used for all downstream binding predictions [27].

### Peptide generation from somatic variants and fusions

**SNV/indel-derived peptides:** For each nonsynonymous somatic variant passing filters, the corresponding mutant protein sequence was reconstructed based on VEP annotation. A sliding window approach encompassing all possible 8- to 11-mers containing the mutation centered on the mutated residue was extracted where possible (shorter for events near termini). From this window, all overlapping 8-mer to 11-mer peptides that included the mutant residue were generated, as these lengths account for the majority of MHC class I ligands. Frameshift and in-frame indels were handled similarly, using the mutant reading frame downstream of the variant to generate overlapping 8–11-mers from the altered region. **Fusion-derived peptides:** For each in-frame gene fusion, the predicted chimeric coding sequence spanning the fusion junction was obtained from Arriba’s annotation. This junction region was translated, and all possible 8–11-mer peptides that span the breakpoint (i.e., include amino acids from both fusion partners) were generated. Only peptides that included the junction were retained, ensuring that fusion-derived neoantigens were strictly non-self.

### MHC binding prediction and neoantigen prioritisation

Peptide–MHC class I binding was predicted using NetMHCpan-4.2, using each patient’s OptiType-inferred HLA-A/B/C alleles. For each peptide–HLA pair, NetMHCpan reported an affinity rank percentile; peptides with rank < 2.0% were prioritized. Peptides with binding > 2.0% were not strictly excluded but heavily penalized by the downstream logistic scoring function, effectively down-weighting them in prioritization. Specifically, a MuPeXI logistic transformation was applied to the NetMHCpan %Rank score: $L\left( x \right) = \frac{1}{{1 + e^{{\left\{ {5\left( {x - 2} \right)} \right\}}} }}$

where 2% is the inflection point, such that strong binders with low %Rank receive values close to 1 and peptides above 2% are rapidly down-weighted. To prioritize candidate neoantigens , a MuPeXI-adapted multi-modal scoring framework was implemented in Python (adapted for Python 3.11 and NetMHCpan-4.2) [28]:

Binding affinity component:

- Strong binding: NetMHCpan rank < 0.5%
- Weak binding: 0.5–2.0%
- Peptides outside 2.0% were assigned near-zero priority scores by the logistic function, effectively deprioritizing them from the final candidate list

Expression component:

- Source gene TPM ≥ 1 (gene-level expression from Kallisto).
- Peptides from genes with TPM < 1 were excluded, approximating insufficient antigen supply.

Clonality component:

- Variant VAF was used as a linear weighting factor for clonality, essentially deprioritizing subclonal events in the final ranking
- For fusions, where VAF is not directly measurable from RNA-seq data, normalized fusion abundance (FFPM) was used as a proxy for expression within the scoring framework, and variants were treated as clonal (VAF = 1.0) given their status as pathognomonic drivers

Self-similarity filter:

- Each candidate peptide was checked for exact matches elsewhere in the human reference proteome (Gencode v49), and those with 100% identity to self-peptides were assigned a priority score of zero

### Neoantigen priority score was computed , as a weighted combination of:

- Inverse binding rank (stronger binders score higher)
- Source gene expression was incorporated using a hyperbolic tangent (tanh) transformation, as described in the MuPeXI framework
- VAF (for SNV/indels) or normalized junction read count (for fusions)
- A foreignness component was calculated using an agretopicity index, comparing the predicted binding rank of the mutant peptide with that of its wild-type counterpart.

This score was used to rank SNV- and fusion-derived neoantigens within each tumor, and the top-ranked peptides formed the focus of downstream interpretation.

### Reproducibility & data availability

The entire analysis workflow was implemented in Snakemake (v9.14.5) to ensure reproducibility and scalability across cohorts [30]. Each major step (QC, alignment, variant calling, fusion detection, HLA typing, peptide generation, MHC binding prediction, scoring) is encapsulated as a separate rule with explicit input/output and software environment definitions. The conda environments were pinned to specific tool versions to maximize reproducibility [34]. The pipeline, along with configuration files and environment specifications, is publicly available at [https://github.com/mantaspanos/sarcoma-multiomics-neoantigens].

## Results

### Genomic quality and strategic repurposing of legacy data

Paired WES and RNA-seq data were repurposed to ask whether CKS tumors yield actionable neoantigens despite the technically constrained legacy datasets with low coverage. Despite these technical constraints, high variant allele frequency (VAF) candidate neoantigens could still be identified. Because CKS tumors are notoriously heterogeneous, candidates with low VAF were deprioritized to address the intratumoral heterogeneity. Initial quality control of the WES data revealed a challenging sequencing output. The assay targeted an extensive target area based on the exome panel footprint but achieved a modest mean depth of 29.3 × with a highly uneven coverage distribution (max depth 4,735x). Only 31% of the target territory achieved ≥ 10 × depth, reflecting a substantial capture bias across coding and non-coding regions.

### Uneven capture efficiency and coverage strategy and distinguishing technical bias from biological signal

**Fig. 1:**
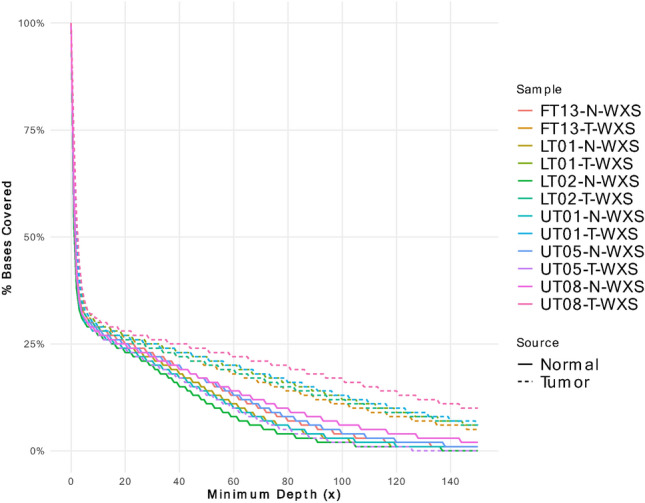
WES QC/coverage: Cumulative breadth-of-coverage plot

The UT05 sample illustrated the problem starkly (Fig. 1) since only 27% of the target remained usable at 10 × depth. To preserve cohort-wide comparability while maintaining mutation sensitivity, the callable territory was thus defined using a ≥ 10 × threshold for downstream analyses. Supplemental Figure S1 shows the depth coverage which indicated that many loci with extreme coverage likely reflect technical capture bias rather than biological amplification, as evidenced by hyper-covered regions shared between tumor and matched normal samples.

**Fig. 2:**
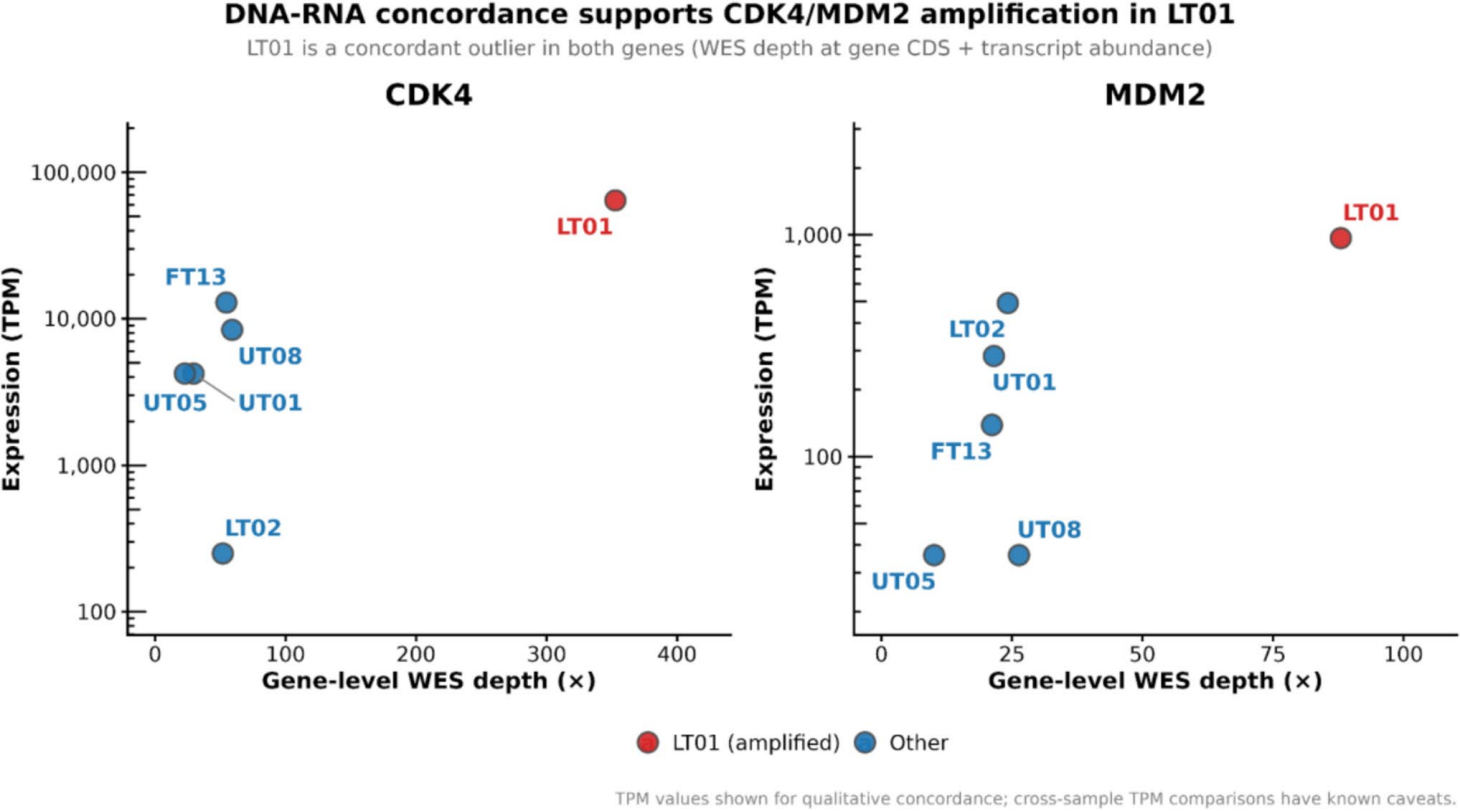
DNA–RNA Concordance at CDK4 and MDM2 as exemplar markers of the LT01 12q Amplicon. Integration of locus-specific WES depth and RNA-seq abundance reveals concordant outliers for both CDK4 and MDM2 in sample LT01, consistent with functional amplification. While other samples show sporadic depth increases (attributable to capture bias), only LT01 exhibits commensurate transcriptional overexpression. TPM values are shown for qualitative concordance not cross-sample quantitative comparison though

To identify candidate copy-number events, selected loci were evaluated for multi-omic concordance. In sample LT01, the CDK4 and MDM2 loci displayed a distinct, concordant outlier profile: elevated locus depth (~ 353 × for CDK4) accompanied by extreme transcriptional abundance (64,344 TPM). This dual signal is consistent with the 12q amplicon-driven phenotype described in the CKS molecular classification (Fig. 2). Gene-level concordance across the broader GISTIC2-derived panel used by Kim et al. is shown in Supplemental Figure S2. In contrast, sporadic depth spikes observed at isolated exons in other samples lacked commensurate RNA overexpression, supporting their classification as artifacts.

To further mitigate the risk of underestimating immunogenicity in this uneven landscape, the tumor mutational burden ( was normalized to the sample-specific callable protein-coding territory (CDS regions with ≥ 10 × coverage). This integrative approach refined the quantitative TMB estimates without materially altering the overall cohort interpretation, by preserving the high-TMB status of FT13 and LT02 while improving sensitivity to low coverage but biologically supported events.

### Molecular stratification into immunogenic archetypes

Consistent with the molecular framework reported by Kim et al. [18], the data suggest a hypermutator subset characterized by elevated nonsynonymous TMB and a transition-enriched SNV spectrum in a subset of cases, suggestive of mismatch repair deficiency-like mutagenesis (Table 1). Within the hypermutator group, however, mutational spectra were heterogeneous: FT13 and UT05 showed markedly elevated Ti/Tv ratios, whereas LT02 remained highly mutated by TMB yet exhibited a distinct Ti/Tv profile, indicating that “hypermutation” in this cohort is not mechanistically uniform. To support downstream neoantigen interpretation without over-attributing biology individually, the following metrics were integrated, i.e., mutation burden (nonsynonymous TMB), mutational spectrum (Ti/Tv as a transparency/QC descriptor), structural variation (expressed fusions), and driver context into two broad immunogenic axes: (i) a hypermutator group (MSI-like or non-MSI-like) and (ii) a low-SNV group with mechanistically distinct neoantigen sources, including desert-like (UT01), fusion-associated (UT08), and amplification-driven (LT01) cases.

**Table 1:** Immunogenomic profile of the cohort: summarizes cohort-level immunogenomic features, including nonsynonymous TMB normalized to sample-specific callable protein-coding CDS territory (≥ 10 ×), Ti/Tv ratios computed from PASS SNVs within capture targets (reported primarily for contextual interpretation and QC), representative driver events, and the distribution of predicted neoepitopes by source (SNV/indel versus fusion). Ti/Tv is sensitive to target definition and filtering stringency; therefore, it is not used as a standalone classifier of MSI status or treatment etiology but rather as supportive context alongside TMB and structural evidence

Sample	Phenotype	TMB	TiTv	Driver events	Neoepitope counts
FT13	Hypermutator (MSI-like)	16.43	4.05	PTEN indel, OBSCN mutation	1088 (1088 SNV / 0 Fusion)
LT02	Hypermutator (chemo-induced, non-MSI)	15.25	2.37	ATRX mutation	933 (892 SNV / 41 Fusion)
UT05	Hypermutator (MSI-like)	6.09	7.54	TP53, PTEN, RB1 mutations	903 (903 SNV / 0 Fusion)
UT08	SNV-low/fusion associated	0.83	1.42	NF1::BLTP3B, KDM2A::MYH9 fusions	65 (58 SNV / 7 Fusion)
UT01	SNV-low / No canonical driver	0.90	1.79	No recurrent driver identified (SNV/indel)	83 (83 SNV / 0 Fusion)
LT01	SNV-low / amplification-driven	0.64	2.54	CDK4/MDM2 amplicon	37 (24 SNV / 13 Fusion)

Cross-validation against Kim et al. confirmed that TP53/ATRX/PTEN alterations were observed in hypermutator-classified samples (Supplemental Table S1) and CDK4 amplification in LT01 [18]. This analysis broadens these conclusions by identifying expressed structural variants (NF1::BLTP3B, KDM2A::MYH9) as immunogenic sources in SNV-low contexts (Table S3). Notably, LT02 generated 41 fusion-derived candidates versus zero in naïve MSI-like samples (FT13/UT05), consistent with the possibility that therapy may enrich junction neoantigens with enhanced foreignness potential.

Lastly, Table 1 shows that sample UT08 is fusion-associated despite low-SNV burden, whereas LT01 represents an amplification-driven SNV-low case. Fusion neoepitopes and structural complexity provide complementary immunogenomic sources beyond the SNV burden alone.

### RNA-guided “rescue” identifies expressed target reservoirs

RNA-seq provided orthogonal support for events occurring in genomic regions interpreted with ambiguity, enabling the differentiation of true biological signals from technical or baseline variation. In sample FT13, the PTEN locus exhibited heterogeneous coverage which challenged high-confidence conventional somatic calling; however, transcriptomic evidence supported prioritization of this driver-associated event. Even with poor WES depth, RNA-sequencing confirmed the mutant allele at high transcriptional abundance (107.5 TPM), validating expression of a frameshift (p.Asn323MetfsTer40). This produced a strong-binding neoepitope (YLVLTLTKV; Rank 0.175%), demonstrating that mutational expression can prioritize actionable targets in spite of coverage gaps.

Similarly, the integration of WES depth and RNA abundance effectively validated the functional copy-number drivers. Figure 2 highlights sample LT01 as a distinct outlier, exhibiting simultaneous elevation of genomic coverage and transcript levels for both CDK4 and MDM2 genes. Comparing these syntenic genes strengthened the diagnosis of a functional 12q15 amplification in LT01, separating it from cases such as FT13, where high CDK4 expression occurred without MDM2 co-elevation or genomic gain—a pattern indicative of transcriptional regulation rather than chromosomal amplification. This concordance-based framework supports interpretation of driver-associated events.

### Prioritization of candidate neoepitopes

**Fig. 3:**
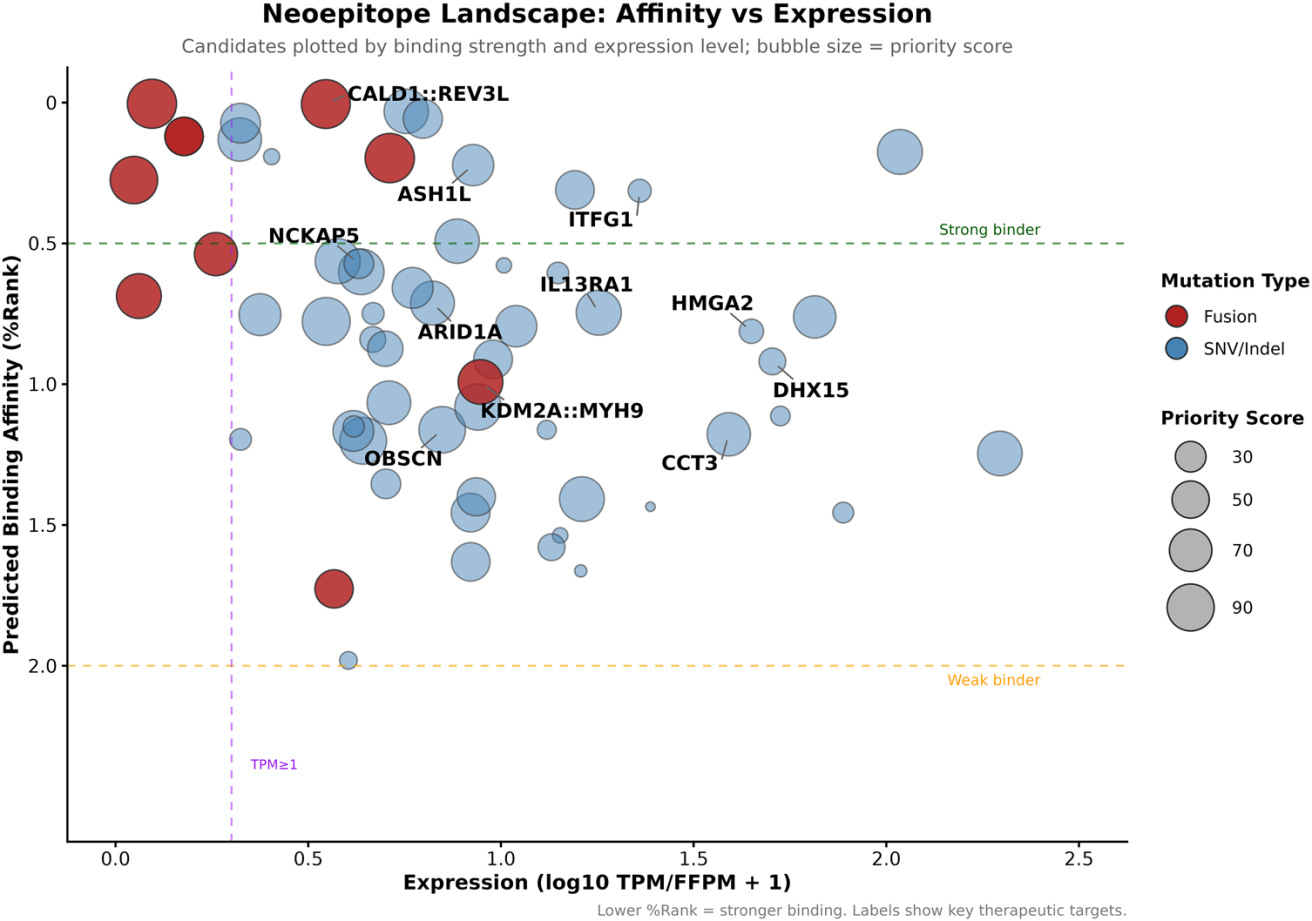
Neoepitope landscape: Predicted MHC binding affinity versus expression level. Each point represents a candidate neoantigen colored by mutation type (SNV/indel: blue; Fusion: red) and sized proportional to the composite priority score. The x-axis shows log_10_ (TPM + 1) for SNV-derived candidates and log_10_ (FFPM + 1) for fusion junctions. Labeled points highlight high-confidence therapeutic candidates across archetypes, including the exceptional CALD1::REV3Lfusion (rank 0.005%) and driver-associated targets (ARID1A, HMGA2). Dashed lines indicate strong-binder (≤ 0.5%) and weak-binder (0.5–2%) thresholds, with the vertical line marking minimum expression (TPM/FFPM ≥ 1). This multi-dimensional prioritization reveals expressed, high-affinity candidates suitable for personalized immunotherapy despite technical constraints in legacy sequencing data.

Candidate prioritization integrated predicted HLA binding strength (NetMHCpan %Rank), expression support (TPM for SNV/indel-derived candidates; FFPM for fusion junctions), and a composite priority score. HLA genotyping confirmed that the allele distribution of the cohort mirrored the high-frequency haplotypes in the Korean population (for example, A*24:02, A*33:03-B*58:01), supporting the relevance of the results to the local demographics (Supplemental Table S2). The expression–affinity landscape (Fig. 3) summarizes the selection space and stresses candidates that combine detectable expression with strong predicted binding.

Notably, fusion-derived candidates contributed significantly to the high-affinity tail in some samples. In LT02, CALD1::REV3L achieved an exceptional predicted binding affinity (0.005% Rank) with detectable fusion abundance, showing the potential of expressed junction peptides to rank alongside or above SNV-derived candidates. In UT08, candidate ranking reflected an abundance-weighted rationale: KDM2A::MYH9 was retained because higher fusion abundance (7.85 FFPM) partially offset intermediate binding rank, whereas lower-abundance junctions were deprioritized.

Table 2 lists a selection of high-priority neoepitopes (extended candidate lists and top five candidates, are provided in Supplemental Table S3). The presented candidates are two per sample and identified through the current multi-omic discovery pipeline. These candidates, spanning both immunogenomic axes and all low-SNV subtypes, demonstrated the capacity of the workflow to prioritize effectively across diverse mutation burdens. High-quality fusion events emerged as leading candidates, with CALD1::REV3L (LT02) achieving an exceptional binding rank of 0.005%, and KDM2A::MYH9 (UT08) demonstrating robust expression support (7.85 FFPM). Furthermore, the prioritization framework, defined in Methods section, successfully linked immunogenicity to known driver biology, highlighting neoepitopes derived from PTEN (FT13) and ARID1A (LT02) alongside highly expressed targets such as HMGA2 (LT01; 43.66 TPM). Crucially, the approach proved effective even in candidates with low-SNV burden, recovering viable targets like IL13RA1 (UT01, desert-like) and NCKAP5 (LT01, amplification-driven) from low-TMB samples by using expression-weighted selection to rescue candidates that could otherwise be overlooked.

**Table 2:** Curated neoepitope candidates presented. Two candidates per sample were selected based on integrated evidence from predicted HLA binding (NetMHCpan %Rank), a composite priority score, and expression support (TPM for SNVs/indels; FFPM for fusions)

Sample	Event type	Gene	HLA	Rank (%)	Priority score	Expression
FT13	SNV/Indel	OBSCN	HLA-B*15:11	1.162	88.16	6.04 TPM
FT13	SNV/Indel	PTEN	HLA-A*02:01	0.175	80.76	107.49 TPM
LT01	SNV/Indel	NCKAP5	HLA-B*07:02	0.572	27.85	3.28 TPM
LT01	SNV/Indel	HMGA2	HLA-B*07:02	0.812	15.85	43.66 TPM
LT02	Fusion	CALD1::REV3L	HLA-B*07:02	0.005	98.93	2.51 FFPM
LT02	SNV/Indel	ARID1A	HLA-C*07:02	0.712	76.58	5.64 TPM
UT01	SNV/Indel	IL13RA1	HLA-A*03:01	0.746	81.66	16.94 TPM
UT01	SNV/Indel	DHX15	HLA-A*24:02	0.919	20.78	49.62 TPM
UT05	SNV/Indel	CCT3	HLA-A*30:11	1.178	73.79	38.02 TPM
UT05	SNV/Indel	ASH1L	HLA-A*30:11	0.222	65.51	7.47 TPM
UT08	Fusion	KDM2A::MYH9	HLA-C*05:01	0.992	47.73	7.85 FFPM
UT08	SNV/Indel	ITFG1	HLA-B*48:01	0.313	13.42	21.91 TPM

TPM, transcripts per million; FFPM, fusion fragments per million. NetMHCpan %Rank thresholds:

< 0.5, strong binder; 0.5–2 weak binder.

To assess generalizability, the workflow was additionally applied to the remaining matched cases from the original study and to independent validation datasets; metadata and neoepitope summary results for the extended cohort are provided in the Supplemental Materials (Fig. S3 and Table S4).

The fusion neoantigen module was independently validated using publicly available RNA-seq data from two well-characterized Ewing sarcoma cell lines, A673 and TC71 (GSE132966). In both datasets, the pipeline autonomously identified the hallmark EWSR1::FLI1 fusion as the top-ranked fusion candidate, with junction-spanning peptides achieving NetMHCpan binding ranks of < 0.5% and priority scores of 99.98 and 99.20, respectively. These results support the performance of the fusion detection and neoepitope prioritization module on biologically validated, independent datasets. Note that this validation applies specifically to the fusion-detection component; SNV-derived neoantigen calling was not benchmarked in cell line models, as this requires matched normal DNA which was not available for these public datasets.

## Discussion

Legacy WES and RNA-seq sarcoma cohorts are still valuable, but the technical constraints, i.e., large target footprints, uneven capture, and variable depth, complicate standard immunogenomic readouts. In CKS, chromosomal chaos and subclones make it worse. The present analysis indicates that a conservative callability definition coupled with multi-omic concordance can recover interpretable immunogenomic structures from such datasets, while reducing reliance on any single metric.

These results also support a stratified view of immunogenic potential. Hypermutator cases yield a large number of SNV/indel-derived candidates, but high candidate counts alone are not sufficient for downstream translational relevance in heterogeneous tumors. Conversely, SNV-low tumors can still produce a limited number of high-priority candidates through expressed structural events or potentially via expression-supported prioritization in low-SNV tumors. This observation is consistent with CKS being driven largely by copy-number alterations and structural complexity, including CDK4-associated subtypes described previously.

Methodologically, isolated extreme WES depth is not sufficient evidence of amplification in legacy capture data. The tumor–normal depth dispersion suggests capture bias contributes substantially to these spikes, so we treated DNA–RNA concordance as the deciding evidence for functional amplification. The sample LT01 (CDK4/MDM2 concordant outlier co-elevation) is a concrete example of how RNA helps resolve ambiguous DNA-only signals in uneven datasets.

The analysis also emphasizes the essential role of orthogonal transcriptomic evidence in rescuing driver events that may be obscured by heterogeneous genomic capture. The PTEN frameshift in sample FT13 serves as a prime example: despite variable DNA coverage across the gene locus which challenged high-confidence allele counting, RNA-seq provided decisive validation. By confirming the robust expression of the mutant transcript (107.5 TPM), this event could be confidently prioritized as a functional neoantigen-generating driver, bypassing the ambiguity of the WES data. This demonstrates that RNA-seq acts not simply as a validation layer, but as an essential discovery tool for resolving actionable targets in regions of suboptimal genomic capture.

This study has several limitations. Candidate lists are computational predictions and do not establish immunogenicity or in vivo presentation; empirical confirmation (e.g., peptide–MHC presentation by immunopeptidomics and functional T cell assays) is required to confirm immune recognition. In addition, the cohort size was small and reflected a particular legacy dataset, limiting the extrapolation across sarcoma histologies and sequencing protocols. Nevertheless, the analytic pattern, QC-aware callability, concordance-based driver interpretation, and expression-informed prioritization, should be transferred to other archived multi-omic cohorts.

Overall, the analysis endorses a practical workflow for extracting interpretable immunogenomic signals and prioritized candidate lists from uneven legacy WES/RNA-seq data in genomically complex sarcomas, complementing established CKS molecular stratification based on copy-number and expression signatures.

## Supplementary Information

Below is the link to the electronic supplementary material.

Supplementary file1 (DOCX 1444 kb)

## Data Availability

Raw sequencing data are available via ENA accession PRJEB23898 and PRJEB24352.

## References

[1] Anzar I et al (2023) The interplay between neoantigens and immune cells in sarcomas treated with checkpoint inhibition. Front Immunol 14:1226445. 10.3389/fimmu.2023.1226445 37799721 10.3389/fimmu.2023.1226445 PMC10548483

[2] Kiyotani K, Chan HT, Nakamura Y (2018) Immunopharmacogenomics towards personalized cancer immunotherapy targeting neoantigens. Cancer Sci 109(3):542–549. 10.1111/cas.13498 29288513 10.1111/cas.13498 PMC5834780

[3] Capietto A-H, Hoshyar R, Delamarre L (2022) Sources of cancer neoantigens beyond single-nucleotide variants. Int J Mol Sci 23(17):10131. 10.3390/ijms231710131 36077528 10.3390/ijms231710131 PMC9455963

[4] Crompton BD et al (2014) The genomic landscape of pediatric Ewing Sarcoma. Cancer Discov 4(11):1326–1341. 10.1158/2159-8290.CD-13-1037 25186949 10.1158/2159-8290.CD-13-1037

[5] Tirode F et al (2014) Genomic landscape of Ewing Sarcoma defines an aggressive subtype with co-association of STAG2 and TP53 mutations. Cancer Discov 4(11):1342–1353. 10.1158/2159-8290.CD-14-0622 25223734 10.1158/2159-8290.CD-14-0622 PMC4264969

[6] Siozopoulou V et al (2021) Immune checkpoint inhibitory therapy in sarcomas: Is there light at the end of the tunnel? Cancers 13(2):360. 10.3390/cancers13020360 33478080 10.3390/cancers13020360 PMC7835811

[7] Perry JA et al (2014) Complementary genomic approaches highlight the PI3K/mTOR pathway as a common vulnerability in osteosarcoma. Proceed National Acad Sci 111(51):E5564–E5573. 10.1073/pnas.1419260111 10.1073/pnas.1419260111 PMC4280630 25512523

[8] Song Y, Yang K, Sun T, Tang R (2021) Development and validation of prognostic markers in sarcomas base on a multi-omics analysis. BMC Med Genomics 14(1):31. 10.1186/s12920-021-00876-4 33509178 10.1186/s12920-021-00876-4 PMC7841904

[9] Statz-Geary K et al (2025) DNA damage repair pathway alterations and immune landscape differences in pediatric/adolescent, young adult (AYA) and adult sarcomas. Cancers 17(12):1962. 10.3390/cancers17121962 40563612 10.3390/cancers17121962 PMC12191036

[10] Keskin DB et al (2019) Neoantigen vaccine generates intratumoral T cell responses in phase Ib glioblastoma trial. Nature 565(7738):234–239. 10.1038/s41586-018-0792-9 30568305 10.1038/s41586-018-0792-9 PMC6546179

[11] Li J et al (2023) The screening, identification, design and clinical application of tumor-specific neoantigens for TCR-T cells. Mol Cancer 22(1):141. 10.1186/s12943-023-01844-5 37649123 10.1186/s12943-023-01844-5 PMC10466891

[12] Luo Z et al (2021) Self‐adjuvanted molecular activator (SeaMac) nanovaccines promote cancer immunotherapy. Adv Healthc Mater 10(7):2002080. 10.1002/adhm.202002080 10.1002/adhm.202002080 33336537

[13] Shi W et al (2023) Advances in tumor antigen‐based anticancer immunotherapy: recent progress, prevailing challenges, and future perspective. Adv Ther 6(2):2200239. 10.1002/adtp.202200239

[14] Xie N, Shen G, Gao W, Huang Z, Huang C, Fu L (2023) Neoantigens: promising targets for cancer therapy. Signal Transduct Target Ther 8(1):9. 10.1038/s41392-022-01270-x 36604431 10.1038/s41392-022-01270-x PMC9816309

[15] Sha H et al (2022) Case report: pathological complete response in a lung metastasis of phyllodes tumor patient following treatment containing peptide neoantigen nano-vaccine. Front Oncol 12:800484. 10.3389/fonc.2022.800484 35211402 10.3389/fonc.2022.800484 PMC8861377

[16] Seki M et al (2015) Integrated genetic and epigenetic analysis defines novel molecular subgroups in rhabdomyosarcoma. Nat Commun 6(1):7557. 10.1038/ncomms8557 26138366 10.1038/ncomms8557 PMC4506514

[17] Shevkoplias A et al (2025) Molecular subtyping and insights into sarcoma biology and prognosis. J Clin Oncol 43(16_suppl):11536–11536. 10.1200/JCO.2025.43.16_suppl.11536

[18] Kim J et al (2018) Integrated molecular characterization of adult soft tissue sarcoma for therapeutic targets. BMC Med Genet 19(S1):216. 10.1186/s12881-018-0722-6 30598078 10.1186/s12881-018-0722-6 PMC6311917

[19] S. Andrews, FastQC: A quality control tool for high throughput sequence data. (2010). [Online]. Available: http://www.bioinformatics.babraham.ac.uk/projects/fastqc

[20] Martin M (2011) Cutadapt removes adapter sequences from high-throughput sequencing reads. EMBnetjournal 17(1):10. 10.14806/ej.17.1.200

[21] Li H, Durbin R (2009) Fast and accurate short read alignment with Burrows–Wheeler transform. Bioinformatics 25(14):1754–1760. 10.1093/bioinformatics/btp324 19451168 10.1093/bioinformatics/btp324 PMC2705234

[22] Mudge JM et al (2025) GENCODE 2025: reference gene annotation for human and mouse. Nucleic Acids Res 53(D1):D966–D975. 10.1093/nar/gkae1078 39565199 10.1093/nar/gkae1078 PMC11701607

[23] Dobin A et al (2013) STAR: ultrafast universal RNA-seq aligner. Bioinformatics 29(1):15–21. 10.1093/bioinformatics/bts635 23104886 10.1093/bioinformatics/bts635 PMC3530905

[24] Bray NL, Pimentel H, Melsted P, Pachter L (2016) Near-optimal probabilistic RNA-seq quantification. Nat Biotechnol 34(5):525–527. 10.1038/nbt.3519 27043002 10.1038/nbt.3519

[25] McKenna A et al (2010) The genome analysis toolkit: a MapReduce framework for analyzing next-generation DNA sequencing data. Genome Res 20(9):1297–1303. 10.1101/gr.107524.110 20644199 10.1101/gr.107524.110 PMC2928508

[26] Uhrig S et al (2021) Accurate and efficient detection of gene fusions from RNA sequencing data. Genome Res 31(3):448–460. 10.1101/gr.257246.119 33441414 10.1101/gr.257246.119 PMC7919457

[27] Szolek A, Schubert B, Mohr C, Sturm M, Feldhahn M, Kohlbacher O (2014) OptiType: precision HLA typing from next-generation sequencing data. Bioinformatics 30(23):3310–3316. 10.1093/bioinformatics/btu548 25143287 10.1093/bioinformatics/btu548 PMC4441069

[28] Bjerregaard A-M, Nielsen M, Hadrup SR, Szallasi Z, Eklund AC (2017) MuPeXI: prediction of neo-epitopes from tumor sequencing data. Cancer Immunol Immunother 66(9):1123–1130. 10.1007/s00262-017-2001-3 28429069 10.1007/s00262-017-2001-3 PMC11028452

[29] Mölder F et al (2021) Sustainable data analysis with Snakemake. F1000Res 10:33. 10.12688/f1000research.29032.1 34035898 10.12688/f1000research.29032.1 PMC8114187

[30] Anaconda Software Distribution. (Nov. 2016). [Online]. Available: https://anaconda.com.

