# Correction: Repurposing public sarcoma multi-omics for neoantigen discovery

**DOI:** 10.1007/s00262-026-04469-x

**Published:** 2026-07-04

**Authors:** Panagiotis Mantas, Karen A. Krogfelt

**Affiliations:** 1 Department of Health Technology, Technical University of Denmark, 2800 Kongens Lyngby, Denmark; 2 Department of Science and Environment, Roskilde University, 4000 Roskilde, Denmark

**Correction to: Cancer Immunology, Immunotherapy (2026) 75:152** 10.1007/s00262-026-04395-y.

In the original version of this article, Fig. 3 misses the colours. The Fig. 3 should have appeared as shown below.

**Fig. 3:**
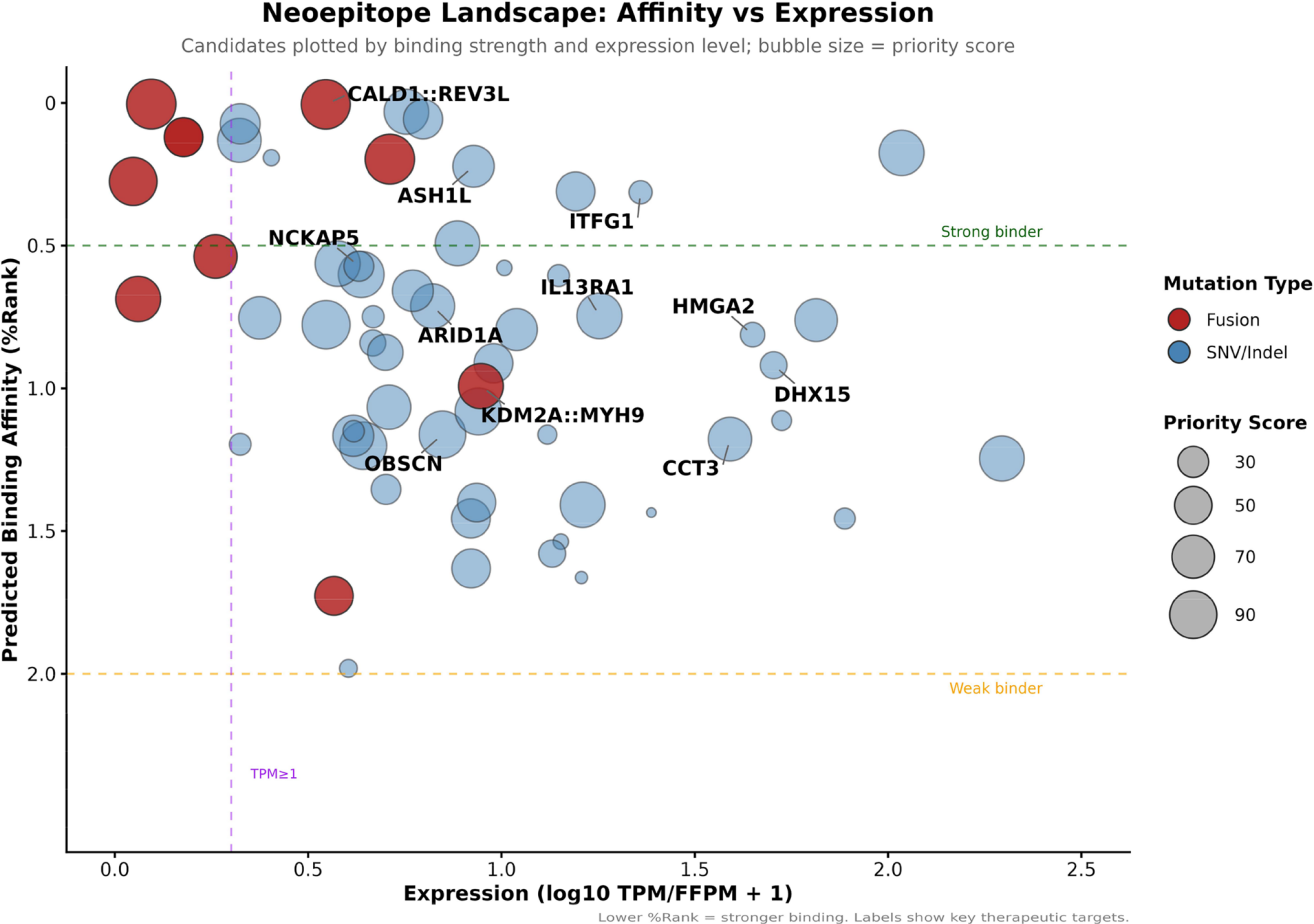
Neoepitope landscape: Predicted MHC binding affinity versus expression level. Each point represents a candidate neoantigen colored by mutation type (SNV/indel: blue; Fusion: red) and sized proportional to the composite priority score. The x-axis shows log10 (TPM + 1) for SNV-derived candidates and log10 (FFPM + 1) for fusion junctions. Labeled points highlight high-confidence therapeutic candidates across archetypes, including the exceptional CALD1::REV3Lfusion (rank 0.005%) and driver-associated targets (ARID1A, HMGA2). Dashed lines indicate strong-binder (≤ 0.5%) and weak-binder (0.5–2%) thresholds, with the vertical line marking minimum expression (TPM/FFPM ≥ 1). This multi-dimensional prioritization reveals expressed, high-affinity candidates suitable for personalized immunotherapy despite technical constraints in legacy sequencing data

The original article has been corrected.

