# Supplementary material for: Repurposing public sarcoma multi-omics for neoantigen discovery
Source: Cancer Immunol Immunother. 2026 Apr 21;75(5):152. doi: 10.1007/s00262-026-04395-y (PMC13100081; doi:10.1007/s00262-026-04395-y)
Supplement: Supplementary file 1 — Supplementary file1 (DOCX 1444 kb) [file 262_2026_4395_MOESM1_ESM.docx]

Supplemental material


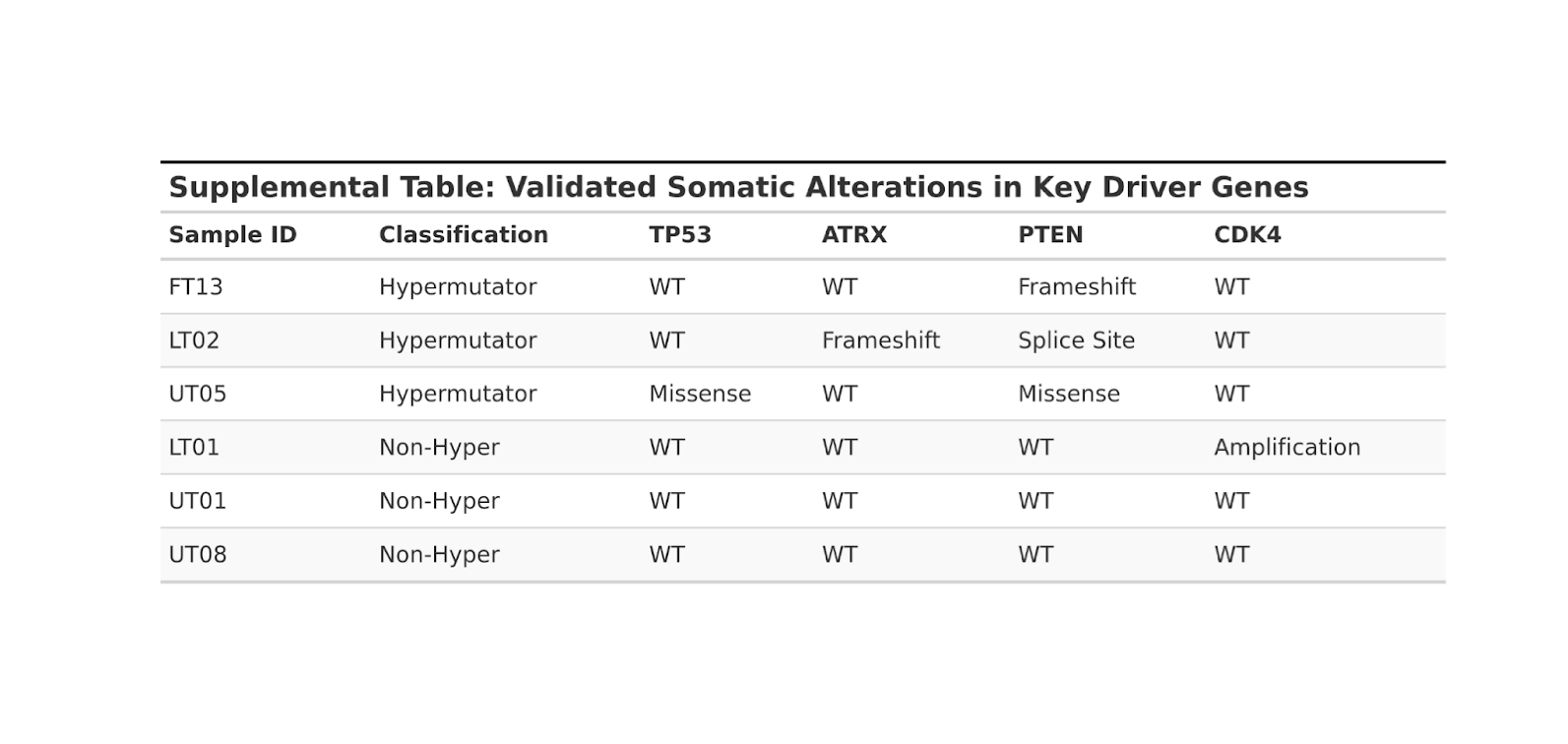


Supplemental Table S1. Verification of somatic driver landscape. Comparison of key driver gene alterations (TP53, ATRX, PTEN, CDK4) detected in this study versus the original publication (Kim et al.). Samples are grouped by hypermutator status. Alteration types (Missense, Frameshift, Splice Site, Amplification) are mentioned; "WT" indicates wild-type status. The observed landscape replicates the enrichment of PTEN/ATRX alterations in hypermutated samples and CDK4 amplification in LT01, confirming pipeline accuracy.


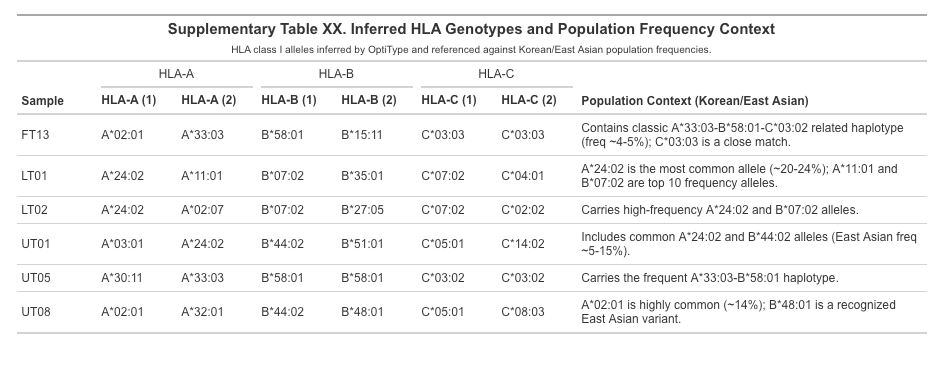


Supplemental Table S2. Inferred HLA Class I Genotypes and Population Context. HLA-A, -B, and -C alleles were determined using OptiType from tumor WXS data. The population context column relates individual genotypes to high-frequency alleles and common haplotypes reported in Korean and East Asian populations, supporting the representative nature of the cohort’s immunogenetic profile.

**
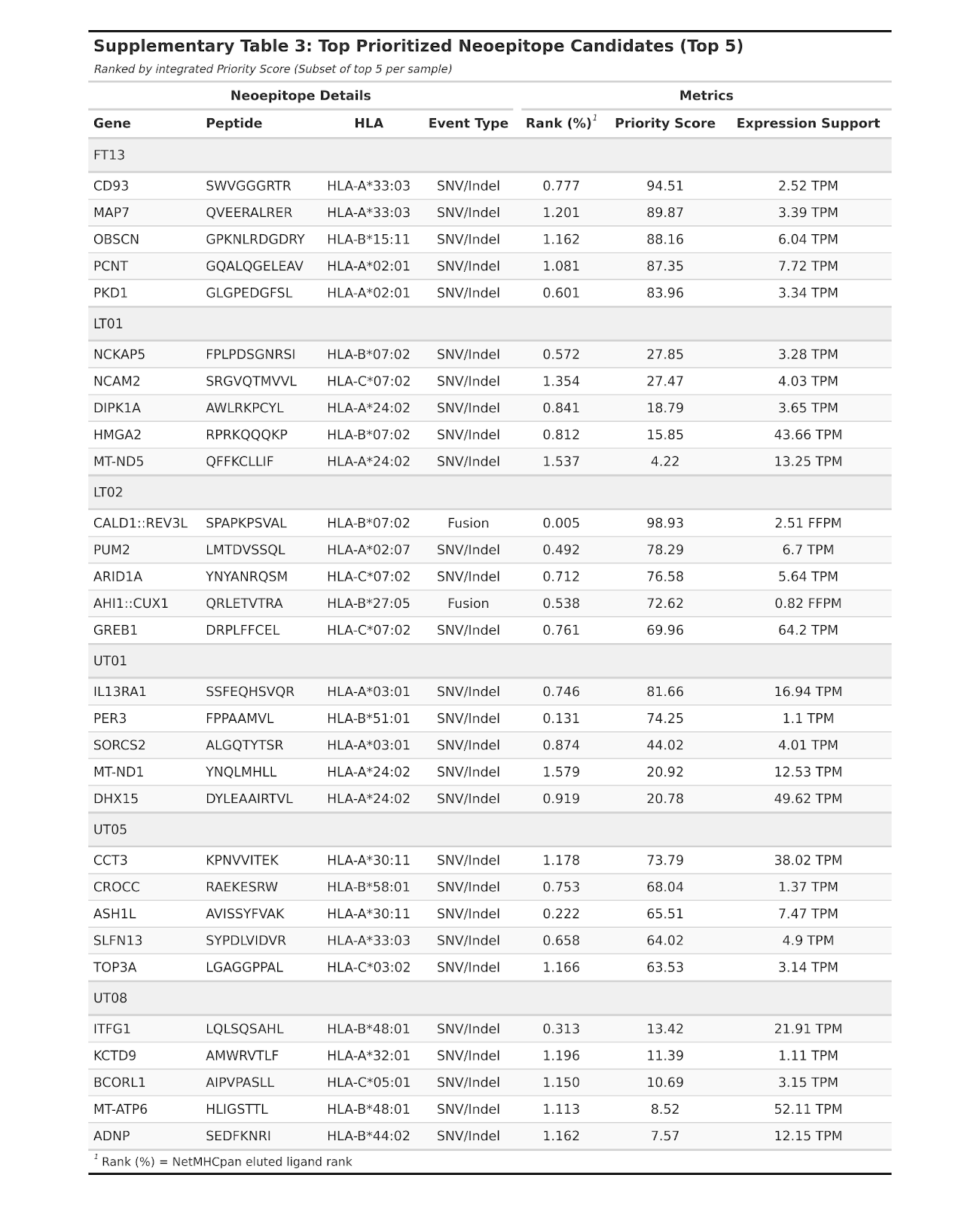
**Supplemental table S3: Top Prioritized Neoepitope Candidates. The top 5 neoepitope candidates per sample were ranked by an integrated Priority Score combining MHC binding affinity, expression support (TPM/FFPM), and agretopicity. Only the highest-scoring peptide per somatic alteration is shown. 'Rank (%)' denotes the NetMHCpan eluted ligand rank.

Supplemental Figure S1. WES coverage: Per-sample mean depth for paired tumor and normal samples with a companion panel showing max depth for the respective samples


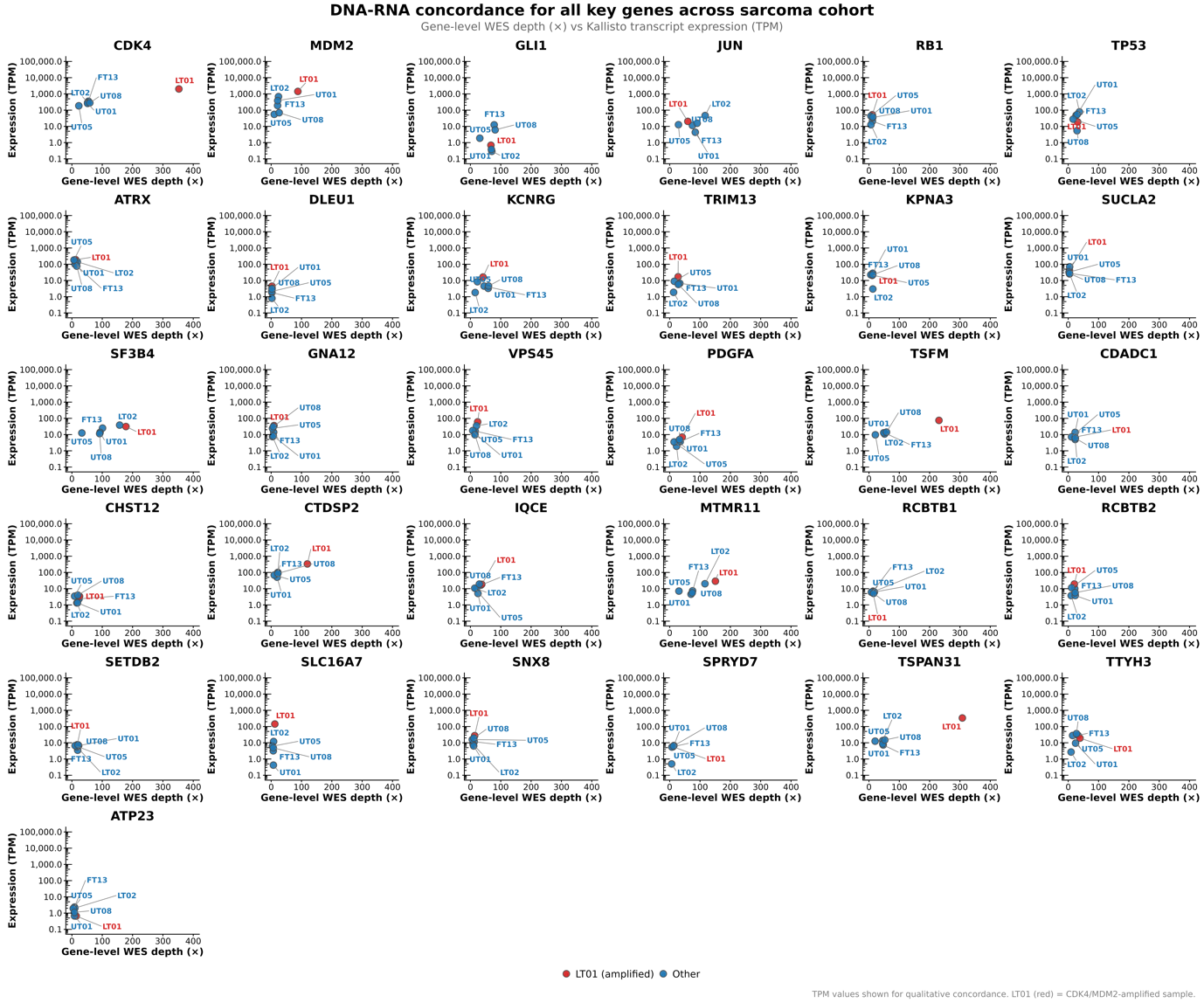


Supplemental Figure S2. Gene-level WES depth versus RNA expression (TPM) for the 26 GISTIC2 peak genes from Kim et al. across the cohort; axes were uniformly scaled across panels.  MDM2 was added manually as the canonical CDK4 co-amplicon, and several additional context genes are shown for visualization of broader DNA–RNA concordance.


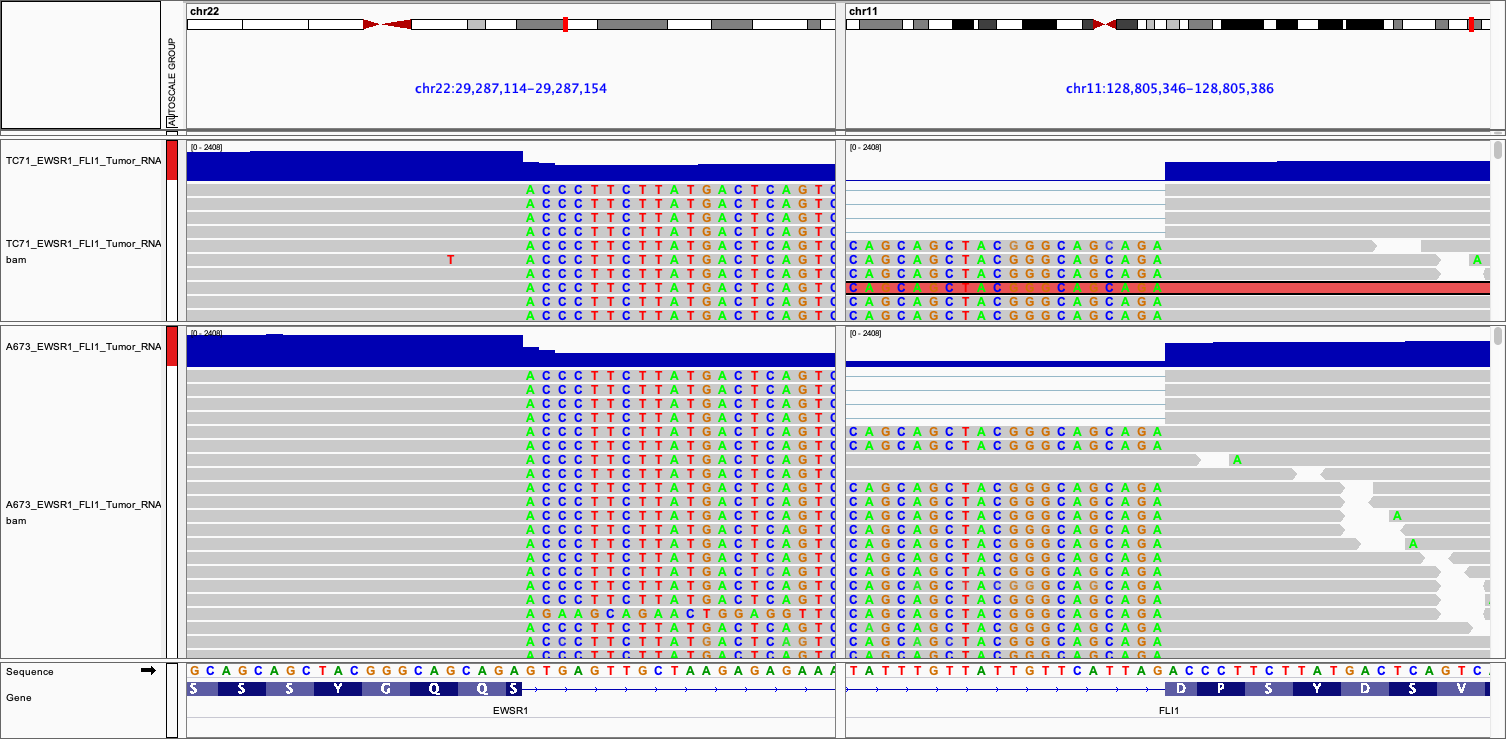


Supplemental Figure S3: Integrated visualization (IGV) of the hallmark EWSR1::FLI1 fusion in validation cell lines. Multi-locus view of RNA-seq alignments for TC71 (top) and A673 (bottom) showing the chromosomal junction between EWSR1 (chr22, left) and FLI1 (chr11, right). Both cell lines show clear junction-spanning reads with characteristic soft-clipping (colored bases) at the exact breakpoint coordinates, confirming hallmark fusion architecture.


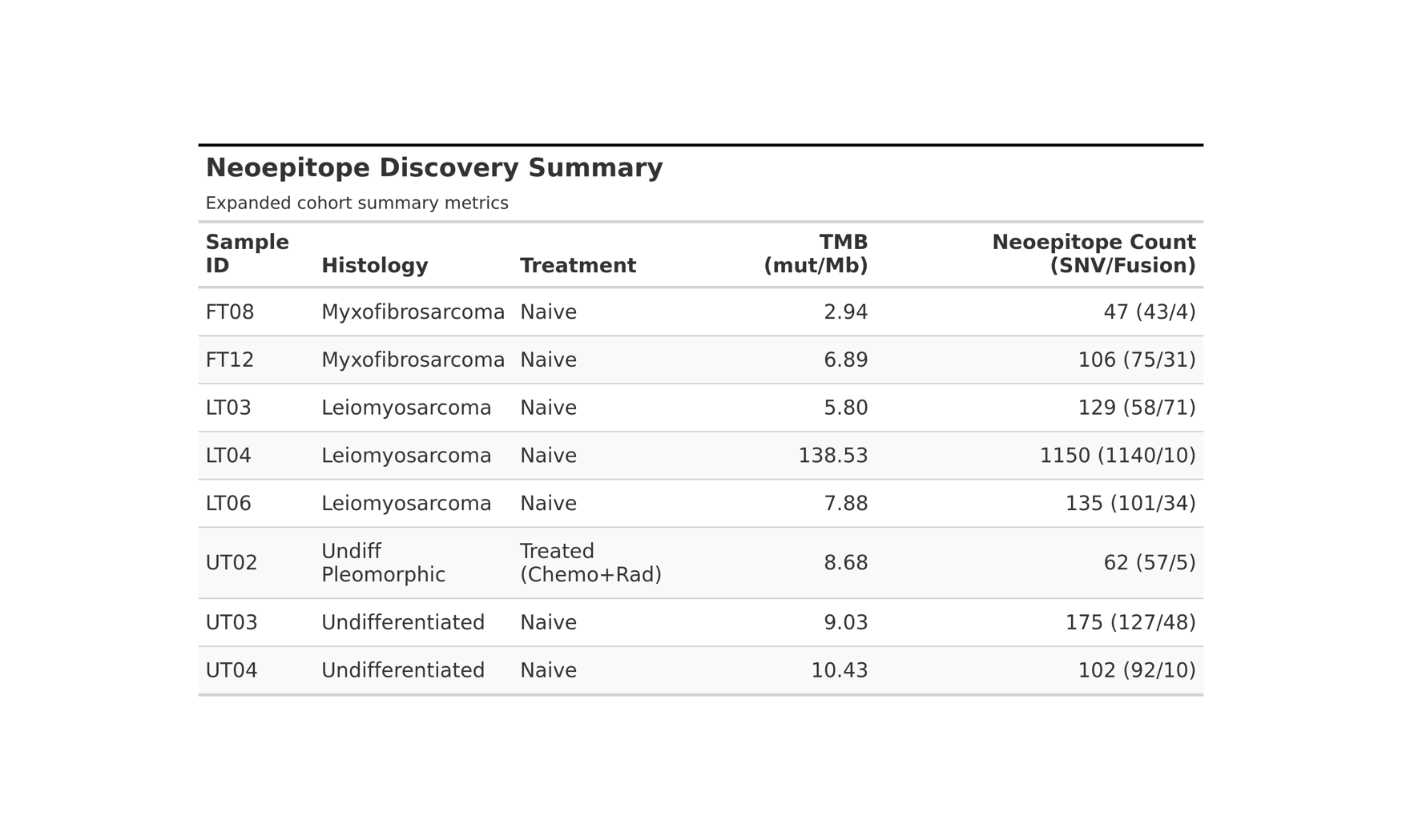


Supplemental Table S4*:* Immunogenomic Profiling and Neoepitope Yield and metadata in the Expanded Cohort.
